# Chondromyxoid Fibroma of the Rib: A Rare Benign Tumor With Potential for Local Recurrence

**DOI:** 10.7759/cureus.19172

**Published:** 2021-10-31

**Authors:** Bappy Basak, Alexander Haragan, Michael Shackcloth, Joyce Thekkudan

**Affiliations:** 1 Department of Cardiothoracic Surgery, Liverpool Heart and Chest Hospital, Liverpool, GBR; 2 Department of Pathology, Royal Liverpool University Hospital, Liverpool, GBR

**Keywords:** long-term follow-up, treatment modalities, malignant potential, rare condition of the rib, chondromyxoid fibroma

## Abstract

Chondromyxoid fibroma (CMF) is a benign cartilaginous tumor that typically occurs in the long bones of young adult males, with the clinical presentation varying from asymptomatic to localized pain, swelling, and movement restriction. We report an unusual presentation of CMF involving a rib, along with a literature review of the management of CMF. Although benign, local recurrence is not uncommon, and malignant transformation has been reported on rare occasions. En bloc surgical excision, with adequate tumor-free resection margins, of radiologically suspected chondromyxoid fibroma is crucial for the treatment and confirmation of diagnosis. A high index of suspicion, adequate treatment, and follow-up are critical for the successful management of these uncommon benign chondroid tumors.

## Introduction

Chondromyxoid fibroma (CMF) accounts for less than 1% of benign bone tumors and consists of myxoid, chondroid, and fibrous tissue [1]. It commonly affects the tibia, and involvement of other sites such as the rib is an unusual presentation [1]. Radiography is essential in identifying and diagnosing this entity and typically is described as an eccentric lesion with a well-defined border of sclerotic bone, although in ribs the lesion may extend throughout the entire length of the affected bone [2]. Calcification is rarely seen [2]. While radiological appearances are almost diagnostic, microscopic evidence of a characteristic lobular architecture composed of bland spindle cells with occasional multinucleated giant cells in a myxoid background is pathognomonic [1,2]. Small biopsies with limited tissue present a diagnostic challenge in these instances. Local recurrence is not uncommon, and rare malignant transformation has also been reported [1-3]. Adequate tumor-free surgical resection margins can help minimize the chances of recurrence [1]. The abstract of this case report was presented as a poster at the Association of Surgeons in Training Annual Conference 2021: Excelling in Adversity on March 5-7, 2021.

## Case presentation

**Figure 1:**
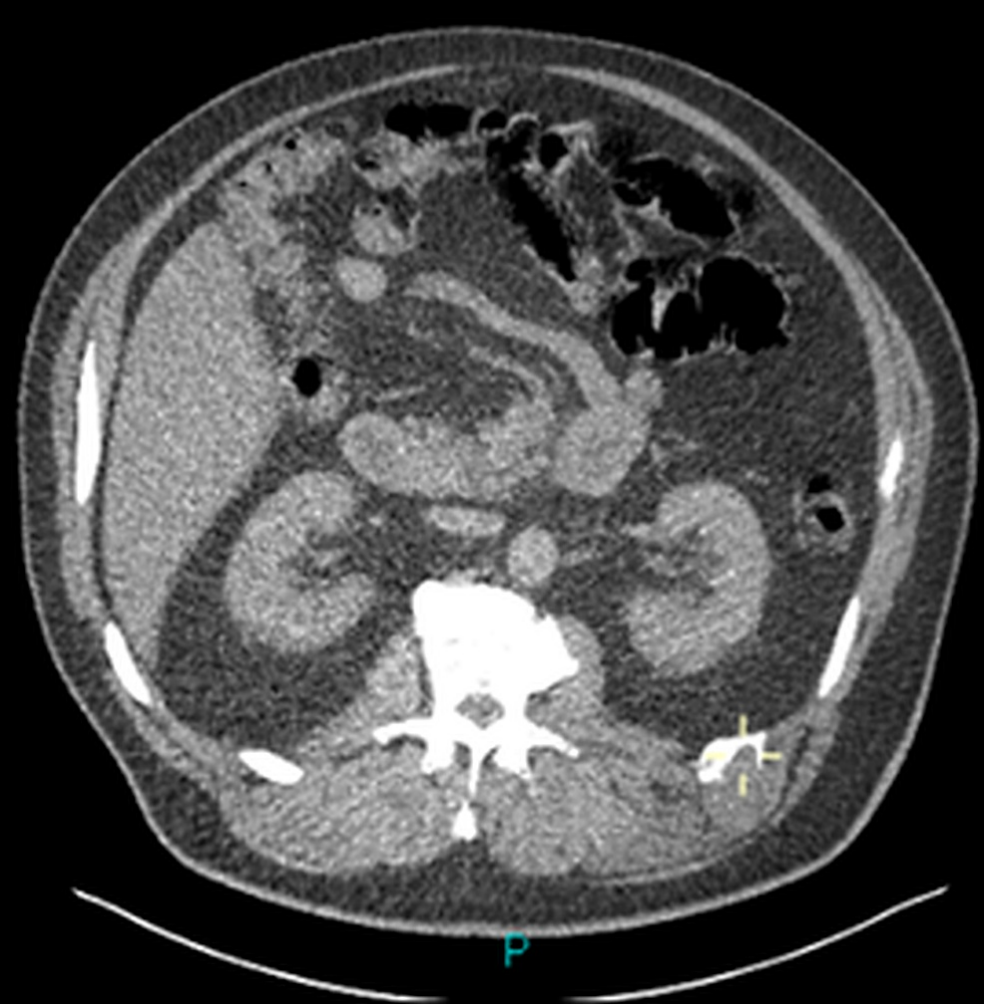
CT scan axial view. CT showing osteolytic lesion in the left 12th rib with the remodeling of the adjacent bone and a lobulated periosteal pattern with mild compression of the adjacent muscles.

**Figure 2:**
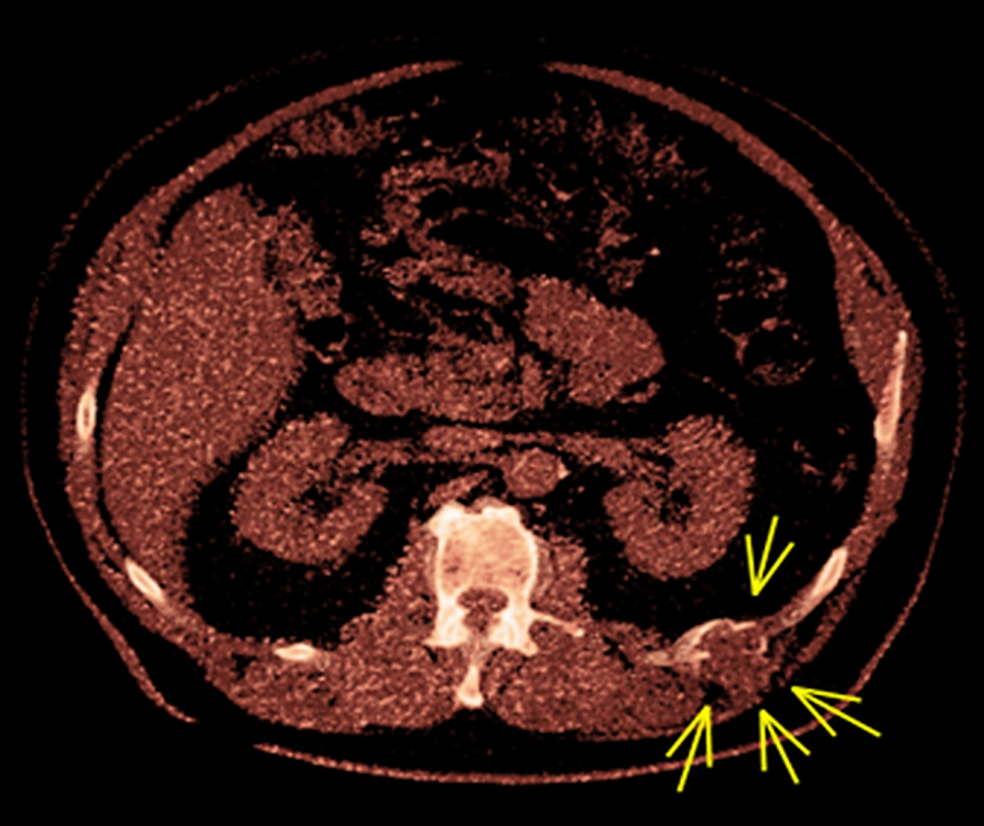
CT scan axial view. CT showing osteolytic lesion in the left 12th rib with the remodeling of the adjacent bone and a lobulated periosteal pattern with mild compression of the adjacent muscles.

**Figure 3:**
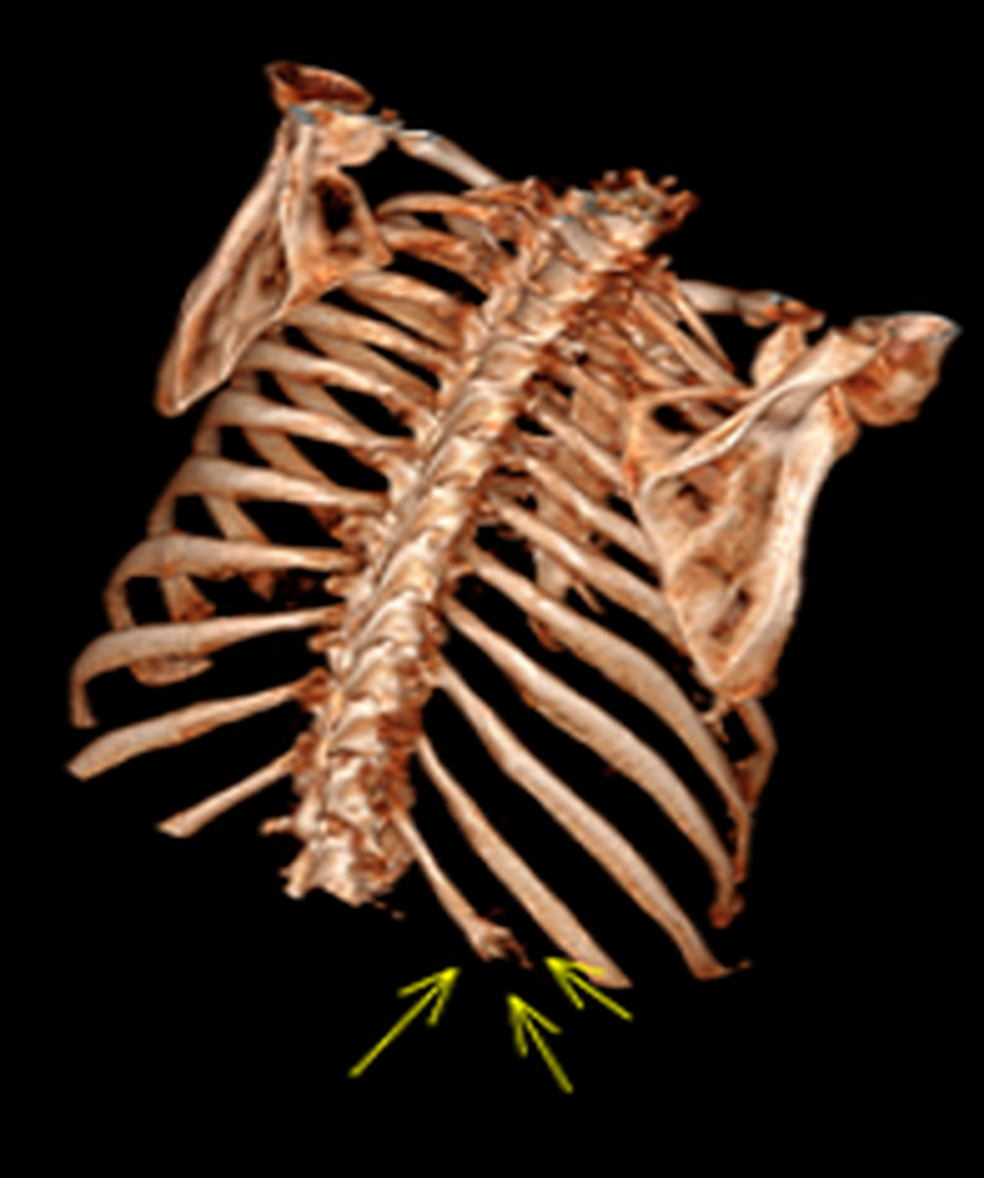
3D reconstruction of the chest wall. Figure showing the lesion on the left 12th rib.

A 61-year-old male was referred with a diagnosis of a benign fibro-osseous lesion based on computed tomography (CT)-guided biopsy, affecting the left 12th rib. He complained of intermittent mild pain and swelling in the left 12th intercostal space. He had no systemic signs or symptoms of malignancy with no significant family history. After a three-year period of radiological stability on CT surveillance, the lesion began to show signs of progression correlating with increased, diffuse tenderness over the costal margin (Figures 1-3). Consequently, the patient agreed to have the rib lesion surgically resected.

**Figure 4:**
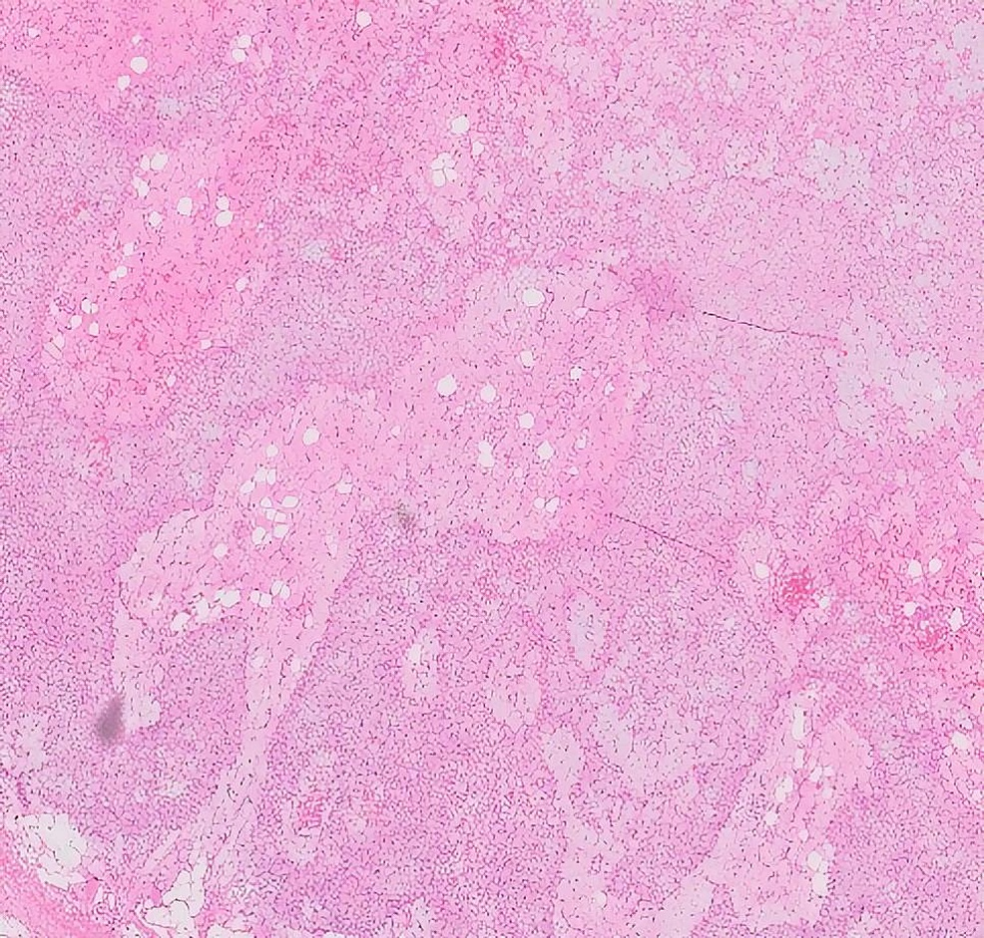
Hematoxylin and eosin staining with classical histological findings, a lobular architecture with intervening bland spindle cells, occasional giant cell, and cartilaginous stroma at varying stages of maturity (10x magnification).

**Figure 5:**
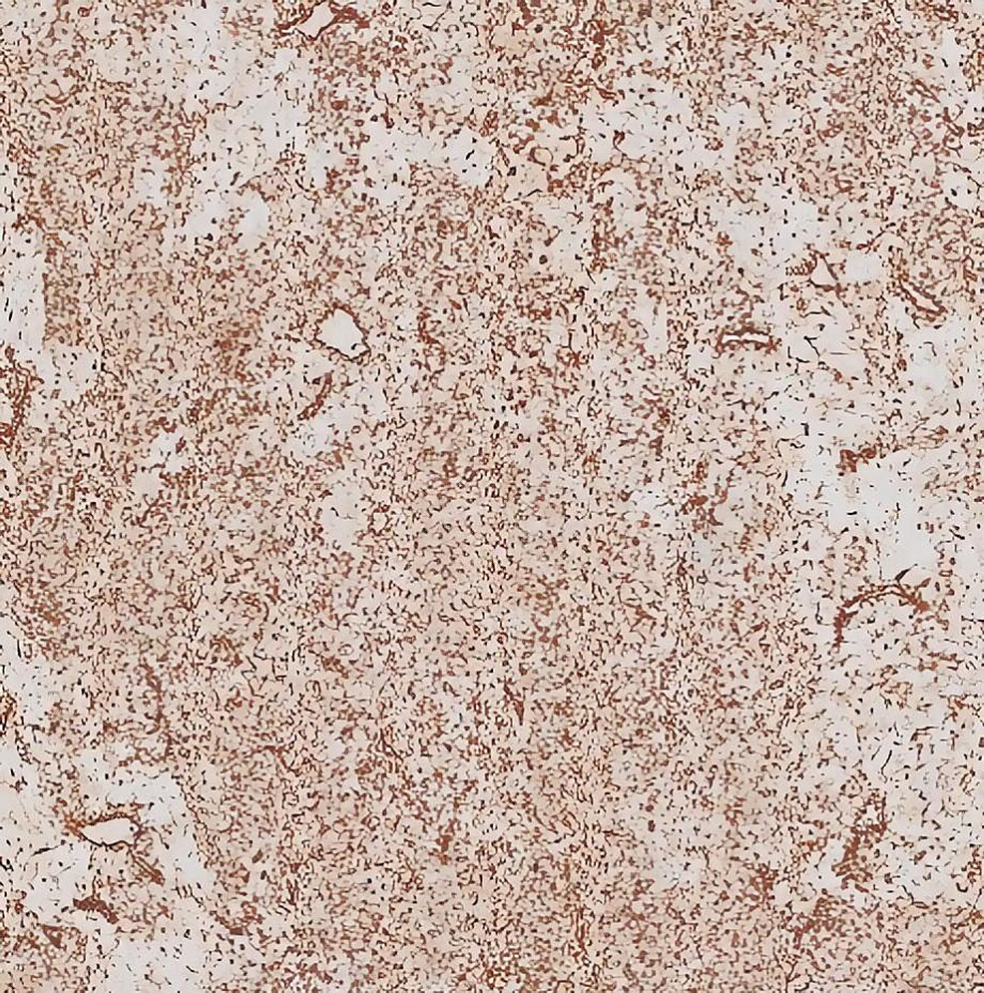
Immunohistochemical staining for vimentin showing diffuse positive staining (10x magnification).

**Figure 6:**
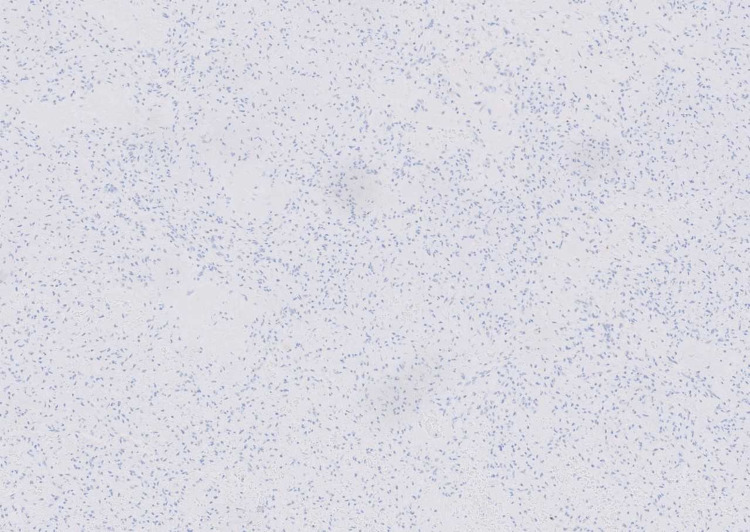
Immunohistochemical staining for pan-cytokeratin demonstrating negative staining of the tumor cells (10x magnification).

He underwent an en bloc resection of the left 12th rib and the surrounding muscle. A left posterior incision was made along the 12th rib. The rib was divided proximally to the tumor with a clear margin of about 1.5 cm. Histopathological examination of the resected material confirmed a chondromyxoid fibroma with clear surgical margins (Figures 4-6). On postoperative follow-up, he developed bulging of the abdominal wall of the left flank, but no true hernia. However, two years after surgery, he remains under CT surveillance with no signs of recurrence on CT scan.

## Discussion

Chondromyxoid fibroma is an uncommon benign tumor of the bone with a relatively high rate of recurrence and a rare risk of malignant transformation. They typically affect the metaphysis of the long bone [1]. They usually present between the ages of 10 and 30 years, with a male predominance [1,2], and with a history of longstanding pain and swelling [1]. Rib presentation is unusual. A case series of 10 cases involving the rib was published in 1972 [1]. Following that, 11 more cases have been reported in different case reports and case series in PubMed so far; however, this is the first case of chondromyxoid fibroma involving the left 12th rib to the best of our knowledge.

Asymptomatic cases are often an incidental diagnosis on radiographs; however, these are not sufficient [4]. CT scan and MRI can give further diagnostic information [4]. Tissue diagnosis can be challenging on small biopsies as evident in our case. A typical biopsy of chondromyxoid fibroma shows lobules of myxoid and chondroid tissue separated by zones of fibrous tissue. The morphological differential diagnoses that need to be ruled out are chondroblastoma, enchondroma, and low-grade chondrosarcoma. Although immunohistochemistry is nonspecific, characteristic histomorphology along with radiological correlation is diagnostic in chondromyxoid fibroma [5].

The treatment options for managing this tumor range from simple curettage to curettage with phenol application, to en bloc excision with bone grafting [6]. Curettage alone has a high recurrence rate of 80% [6], but only 7% recurrence was reported when treated with curettage and bone grafting [2]. A further decrease in recurrence is noted in en bloc excision with bone grafting [2,3]. In our case, the surgical management was an en bloc wide local excision. Due to its location, a bone graft was not considered necessary and could potentially cause a diagnostic dilemma (vis-à-vis recurrence) on surveillance CT scans. Radiotherapy is typically contraindicated as it can lead to potential complications, including malignant transformation [1], although it may be considered in surgically unresectable cases [1]. Recurrence is more common in younger age groups, often associated with higher quantities of myxoid stroma and cytological atypia [1], warranting longer follow-up. Recurrence commonly occurs in the first two years post-resection, but incidences have been reported even after 18 years [4]. Thus, the duration of follow-up remains unclear in the existing literature and, in our view, should be tailored according to patient factors, including age and symptoms, and tumor characteristics (location and resection margins). Considering the local recurrence, our plan is to follow up for five years by CT scans. Due to the diagnostic dilemma and high recurrence rates, chondromyxoid fibroma may be better managed in specialist centers [4].

## Conclusions

Chondromyxoid fibroma is a benign tumor but may demonstrate local involvement of the neighboring tissue and can recur following excision. The diagnosis and management of this uncommon lesion can be challenging. En bloc excision is associated with better long-term outcomes. Careful diagnosis, treatment planning, and adequate follow-up are integral parts of the successful management of this tumor.
